# Extracellular Redox Regulation of α7β Integrin-Mediated Cell Migration Is Signaled via a Dominant Thiol-Switch

**DOI:** 10.3390/antiox9030227

**Published:** 2020-03-10

**Authors:** Lukas Bergerhausen, Julius Grosche, Juliane Meißner, Christina Hecker, Michele F. Caliandro, Christoph Westerhausen, Andrej Kamenac, Maryam Rezaei, Matthias Mörgelin, Gereon Poschmann, Dietmar Vestweber, Eva-Maria Hanschmann, Johannes A. Eble

**Affiliations:** 1 Institute of Physiological Chemistry and Pathobiochemistry, University of Münster, 48149 Münster, Germany; lukas.bergerhausen@googlemail.com (L.B.); julius.grosche@googlemail.com (J.G.); juliane_meissner@gmx.net (J.M.); caliandromichelefabrizio@gmail.com (M.F.C.); mar.rezaei80@gmail.com (M.R.); 2 Department of Neurology, Medical Faculty, Heinrich-Heine University Düsseldorf, 40225 Düsseldorf, Germany; Christina.Hecker@uni-duesseldorf.de (C.H.); eva_hanschmann@web.de (E.-M.H.); 3 Biophysics Group, Department of Experimental Physics, Institute of Physics, University of Augsburg, 86159 Augsburg, Germanyandrej.kamenac@physik.uni-augsburg.de (A.K.); 4 Institute of Theoretical Medicine, University of Augsburg, 86159 Augsburg, Germany; 5 Colzyx AB, 22381 Lund, Sweden; matthias@colzyx.com; 6 Institute of Molecular Medicine I, Functional Redox Proteomics, Heinrich-Heine University Düsseldorf, 40225 Düsseldorf, Germany; gereon.poschmann@hhu.de; 7 Department of Vascular Cell Biology, Max Planck-Institute of Molecular Biomedicine, 48149 Münster, Germany; vestweb@mpi-muenster.mpg.de

**Keywords:** redox regulation, redox signaling, integrin α7β1, thiol-switch, extracellular thioredoxin-1, laminin binding, cell migration

## Abstract

While adhering to extracellular matrix (ECM) proteins, such as laminin-111, cells temporarily produce hydrogen peroxide at adhesion sites. To study the redox regulation of α7β1 integrin-mediated cell adhesion to laminin-111, a conserved cysteine pair within the α-subunit hinge region was replaced for alanines. The molecular and cellular effects were analyzed by electron and atomic force microscopy, impedance-based migration assays, flow cytometry and live cell imaging. This cysteine pair constitutes a thiol-switch, which redox-dependently governs the equilibrium between an extended and a bent integrin conformation with high and low ligand binding activity, respectively. Hydrogen peroxide oxidizes the cysteines to a disulfide bond, increases ligand binding and promotes cell migration toward laminin-111. Inversely, extracellular thioredoxin-1 reduces the disulfide, thereby decreasing laminin binding. Mutation of this cysteine pair into the non-oxidizable hinge-mutant shows molecular and cellular effects similar to the reduced wild-type integrin, but lacks redox regulation. This proves the existence of a dominant thiol-switch within the α subunit hinge of α7β1 integrin, which is sufficient to implement activity regulation by extracellular redox agents in a redox-regulatory circuit. Our data reveal a novel and physiologically relevant thiol-based regulatory mechanism of integrin-mediated cell-ECM interactions, which employs short-lived hydrogen peroxide and extracellular thioredoxin-1 as signaling mediators.

## 1. Introduction

During cell movement and tissue remodeling, cells exert forces onto the extracellular matrix (ECM). Laminins are key components within basement membranes, sheet-like ECM scaffolds, which also surround muscle cells and fibers [1]. Laminin-111 contributes to mechanical force transduction between muscle cells and the ECM [2]. Force-transmitting cell contacts to the laminin network are mediated inter alia via integrins [3,4]. These cell adhesion molecules consist of two subunits, α and β. Their N-terminal extracellular domains jointly form the ligand binding head domain, which is connected via two stalks to the transmembrane and short cytoplasmic domains of each chain [3]. Integrins change between a bent, an extended close and extended open conformation [3,5]. The transition from the bent to the extended conformation is a rotation of the head and stalk domains relative to each other around a pivot. This pivot is formed by peptide loops between the EGF-domains 1 and 2 of the β-subunit and between the thigh and calf-1 domains of the α-subunit. This hinge region of the integrin α subunit contains a pair of cysteines, which, if crosslinked via a disulfide bridge, encompasses a peptide loop of five amino acids. [3]. The bent and extended integrin conformations are distinguished with conformation-dependent antibodies, such as the monoclonal antibody (mAb) 9EG7, whose epitope within the β1 integrin subunit is accessible only in the extended conformation [6,7]. Transition of the extended close to the extended open conformation is a separational move of the stalks of both integrin subunits [3,5]. The extended open conformation binds the ECM ligand with highest activity, while ligand binding activity decreases along the extended close to the bent conformation [3,5]. Binding of ECM ligands to integrins not only anchors the cell mechanically but also transmits environmental cues and signals into the cell [8]. Moreover, integrins are part of a complex regulatory network encompassing other proteins of the adhesion sites and growth factor receptors [9,10].

Four members of the integrin family recognize laminins albeit with isoform-dependent affinities [4]. Integrin α3β1 preferentially binds laminin-322 and laminin-511, but not laminin-111 [11]. In contrast, α6β1, α6β4 and α7β1 bind laminin-111 [12,13]. Integrin α7β1 was first isolated from striated and smooth muscle tissue. Integrin α7 knockout mice suffer from muscle dystrophy and defects in neurite regeneration [14,15]. As an oncogenic marker, integrin α7β1 expression changes during tumorigenesis, such as in glioblastoma [16] and melanoma [17,18].

Integrins and their ECM ligands are targets of reactive oxygen species (ROS) (reviewed in [19,20,21]). In a previous study, we identified six out of about 80 cysteines within integrin α7β1, which were selectively oxidized by hydrogen peroxide [22]. All of them are located within the integrin α7 subunit, two of which are located within the hinge region and four others within the calf-2 domain. Therefore, we hypothesized that integrins are also subject to thiol-based redox regulation. This concept implies the existence of a thiol-switch consisting for instance of a pair of cysteines in close proximity [23]. Along with reversible disulfide bond formation, this cysteine pair changes the protein conformation and activity. Previous studies reported that specific cysteine residues within integrins, located within [24,25,26] and outside [24,27,28,29,30,31] of the α-subunit hinge region, are relevant for integrin function. However, most of these studies did not show their redox-dependent functional reversibility.

The concept of redox regulation implies a pair of oxidizing and reducing agents that forms and splits, respectively, the disulfide bridge within the redox-regulated integrin target. Upon initial contact with laminin-111, cells form redox hot spots in knob-like protrusions, where hydrogen peroxide is produced and the redox potential increases locally and transiently [22]. We surmised that the membrane-permeable hydrogen peroxide activates integrins for ligand binding. Previously, protein disulfide isomerases (PDIs) were reported to redox-modify integrins [27,28]. However, PDIs are isomerases, whereas only oxidoreductases transfer electrons from one molecule to another. Some oxidoreductases, including thioredoxin-1 (Trx1), were shown to be secreted [32,33]. They mediate rapid and reversible reduction of disulfide bonds and serve as key regulators of redox signaling circuits [23,32].

Here, we introduce a new redox circuit that regulates the conformation and ligand binding of α7β1 integrin. We delineate one specific pair of cysteines within the hinge region of the integrin α7 subunit that is oxidized by hydrogen peroxide and reduced by extracellular Trx1, and thus functions as a regulatory thiol switch in a dominant manner.

## 2. Materials and Methods

### 2.1. Cell Culture

Human fibrosarcoma HT1080 cells (ATCC, Manassas, VA, USA) and human embryonic kidney (HEK)-293T cells were cultured in DMEM/high glucose medium (Lonza, Basel, Switzerland) containing 10% (*v/v*) heat-inactivated fetal calf serum (FCS) (Gibco, Waltham, MA, USA), 100 U/mL penicillin and streptomycin (Gibco), at 37 °C and 5% and 7% CO_2_ (for HT1080 and HEK-293T cells, respectively). The HEK-293T cell medium was supplemented with 0.1 mM nonessential amino acids (PAA Laboratories, Cölbe, Germany), 6 mM l-glutamine (Gibco), and 1 mM sodium pyruvate (PAA Laboratories). The Drosophila Schneider’s cell line-2 was grown in SF-900 II SFM medium (Gibco) containing 10% (*v/v*) heat-inactivated FCS.

### 2.2. Mutagenesis of Redox-Modifiable Cysteine Residues within Integrin α7 Subunit

Cysteine-to-alanine mutations in the integrin α7 hinge and calf-2 domains were introduced by MEGAWHOP-PCR. To this end, double-stranded DNA gene blocks (IDT, Skokie, IL, USA) encoding the α7hinge (α7hi) and α7calf-2 (α7ca2) domain-spanning nucleotide sequences 1766–2133 and 3071–3374, respectively, of the mouse integrin ITGA7, splice variant X2, including the mutated codons, were used as primers to replace the relevant cysteines for alanines. Thus, the pBSIISK-vector bearing integrin α7X2 cDNA (kindly provided by Dr. U. Mayer, University of Manchester, UK) [12] was PCR-amplified and mutated into pBSIISK-α7hi and pBSIISK-α7ca2, respectively. The double mutant α7hi-ca2 was generated by amplifying pBSIISK-α7hi with the α7calf-2 gene block. The cDNAs encoding the ITGA7 mutants were subcloned into pCR2.1 vector (Invitrogen, Carlsbad, CA, USA) via their flanking XbaI restriction sites.

### 2.3. Construction of Expression Vectors pIRESneo3-a7 and pUC-hygMT-a7-Fos

The soluble integrin ectodomains were heterodimerized with the zipper domains of Fos and Jun [11,12]. The Fos-zipper-encoding cDNA was PCR-amplified from pUC-hygMT-a3-Fos [11] with the primers, FOS fwd, 5′-CAT CAC CGG TGG GTCGAAC GGG CGG C-3′ and FOS rev2, 5′-CCA CAC CTC CCC CTG AAC C-3′. The amplicon was introduced into pCR2.1-α7 via an AgeI restriction site which had previously been introduced upstream of the transmembrane domain-encoding sequence with directed mutagenesis in an overlap extension PCR with the primers ABM fwd, 5′-AGG AGT ACA TGG CCG TGA AA-3′, and ABM rev, 5′-CAC TAT AGG GCG AAT TGG GC-3′. The mutagenesis primer pair was mut fwd, 5′-AGT CCC CAC CGG TGT CAT CCT CCT GG-3′, and mut rev, 5′-GGA TGA CAC CGG TGG GGA CTC CTT CC-3′ (AgeI site underlined). The first PCRs (primer pairs: AMB fwd and mut rev; mut fwd and ABM rev) yielded 168 and 343 bp fragments, from which a 491 bp amplicon was generated in the second PCR (primer pair: ABM fwd and ABM rev). The entire cDNA insert was subcloned into pUC-hygMT via the flanking XbaI sites. For the full-length constructs, the respective α7 cDNA inserts in the pCR2.1 vector were cut with BssHII, blunted, subsequently cut with XbaI and ligated into the linearized pIRESneo3 vector (ClonTech, Mountain View, CA, USA).

### 2.4. Generation of Stably Transfected HT1080 Cells

In a 24-well-plate, 4 × 10^3^/well HT1080 cells were transfected with 1 µg of SspI-linearized pIRESneo3 constructs encoding full-length α7-cDNAs in 200µl DMEM with 6 µL FuGene6 (Promega, Madison, WI, USA), for 30 min. After 8 h, the supernatant was replaced by DMEM + 10% FCS. Selection started after 48 h, and stable transfectants were cultivated with 400 µg/mL geneticine (Gibco).

### 2.5. Reverse Transcription and Real-Time PCR

RNAs of transfected HT1080 cells were extracted with RNeasy Mini Kit (Qiagen, Hilden, Germany), transcribed via QuantiTect Reverse Transcription Kit (Qiagen) and analyzed for the amount of ITGA7-encoding cDNA by qPCR with RotorGene SYBR Green PCR Kit in the RotorGene Cycler (both Qiagen). The following primers were used: ITGA7-fw, 5′-TTGCTGTTAGCCACGATCAG-3′ and ITGA7-rev: 5′-ATGAAGACATGAGCCCGAAC-3′. Signals were evaluated according to the ΔΔCt-method [34] and normalized to the glyceraldehyde 3-phosphate dehydrogenase (GAPDH) signal. RNA from non-transfected (naïve) HT1080 cells served as negative control.

### 2.6. Flow Cytometry

Naïve and transfected HT1080 cells were harvested and fixed with ice-cold 70% ethanol. They were incubated with blocking buffer (1% BSA/1% horse serum in PBS, pH 7.4, containing 1 mM MgCl_2_ and 2 µg/mL aprotinin), and subsequently for 1.5 h with 1 µg/mL of either mouse anti-α7 antibody (Clone 3C12, Miltenyi, Bergisch Gladbach, Germany) or rat anti-α7 antibody mAb 3518 (Clone #334908, R&D, Minneapolis, MN, USA), or an isotype control antibody (mouse or rat IgG, Sigma Aldrich, Deisenhofen, Germany) in the same buffer. After washing and 1 h incubation with 1 µg/mL phycoerythrin-conjugated secondary antibody in blocking buffer, cells were analyzed in a CyFlow-cytometer with FloMax evaluation software (Sysmex Partec, Münster, Germany).

To quantify the portion of active vs. inactive conformation of β1 integrin, non-fixed HT1080 cells were flow cytometrically analyzed after co-staining with biotinylated rat mAb 9EG7 and mouse mAb MEM-101A (Thermo Fisher Scientific, Waltham, MA, USA) in FACSCelesta (BD Biosciences, San Jose, CA, USA) with the FlowJo V10 software (BD Biosciences). The portion of active integrin β1 was calculated as a ratio of fluorescence signals for each 9EG7- and MEM-101A-stained cell.

### 2.7. Immunofluorescence Staining of Transfected HT1080 Cells

HT1080 cells were seeded on 8-well chamber glass slides (ibidi, Martinsried, Germany), coated with either 10 µg/mL laminin-111 (a gift of Dr. R. Timpl, MPI Martinsried, Germany) or rat-tail collagen I (Gibco), and incubated at 37 °C and 5% CO_2_ for 1 h. After washing with PBS, fixation with 4% formaldehyde for 10 min, blocking with blocking buffer (2% horse serum in PBS) and permeabilization with 0.2% triton X-100, cells were stained with a mouse anti-integrin α7 antibody, Clone 3C12 (1:100) in blocking buffer, at 4 °C, overnight. After being washed, cells were incubated with a secondary Alexa 488-conjugated anti-mouse IgG- antibody (1:1000) (Gibco). F-actin and nuclei were stained with Alexa 568-conjugated phalloidin (1:500) (Gibco) and Hoechst dye (1:1000), respectively, for 1 h and 2 min. After mounting with mounting medium (Dako, Hamburg, Germany) images of specimens were taken with a confocal microscope LSM800 with an oil immersion 40x objective (Zeiss, Oberkochen, Germany).

### 2.8. Transduction of LifeAct GFP into HT1080 Cells and Life Cell Imaging

The lentiviral pLenti-LifeAct-GFP construct (a kind gift from Dr. S. Huveneers, AMC, Amsterdam, Netherlands) was packaged into lentivirus in HEK-293T cells, using second-generation lentiviral packaging plasmids, pMDG.2 and psPAX2 (both are kind gifts from Dr. D. Trono, Addgene plasmids #12259 and #12260), with GeneJammer transfection reagents (Agilent, Waldbronn, Germany). Lentivirus containing supernatant was harvested 72 h after transfection, concentrated with Lenti-X concentrator (Clontech, Saint-Germain-en-Laye, France) and used to transduce HT1080 cells with 10 µg/mL polybrene. Cells were selected with 1 µg/mL puromycin (Toku-E, Sint-Denijs-Westrem, Belgium). After 72 h, cells were seeded to 8-well plastic µ-slide (ibidi) coated with laminin-111 or collagen I (each at 10 µg/mL) and incubated at 37 °C and 5% CO_2_ for 1 h. Adhesion and spreading were recorded with video confocal microscopy in a LSM800, at 37 °C, with 10× and 40× immersion oil objectives, with one z-stack picture/min for 10 min. For each condition, at least 30 cells were imaged.

### 2.9. Production, Purification and Binding Test of Soluble Integrin α7β1

Schneider’s cell line-2 cells were stably co-transfected with pUC-hygMT-α7-Fos (wild type or mutant variants) and pUC-hygMT-β1-Jun [11]. Transfected cells were selected with hygromycin B (Merck, Darmstadt, Germany) and subcloned, and their supernatants were tested in sandwich-ELISA with mAB13 (Becton-Dickenson, Franklin Lakes, USA) and a rabbit anti-integrin β1-antiserum [12], as capturing and detecting antibodies, respectively. A single cell clone was expanded, and cell cultures for production were upscaled to 6 L in a Labfors 5 pilot scale bioreactor (Infors, Bottmingen, Switzerland). Culture conditions were 26 °C, pH 6.2 and pO2 of 50%. Soluble recombinant integrins were prepared as described previously [12,22]. The binding activity of isolated integrin α7β1, with or without prior incubation with 100 µM H_2_O_2_ (Carl Roth GmbH, Karlsruhe, Germany), was analyzed in a titration-ELISA [22] and evaluated as published previously [35].

### 2.10. AFM Measurement of Intermolecular Forces between Soluble Integrin α7β1 and Laminin-111

Freshly cleaved mica (V1, diameter 20 mm, Plano GmbH, Germany) were coated with 3 µg/mL laminin-111. Cantilevers (MSCT, Bruker AFM Probes International), with a spring constant of 23 pN/nm, were amino-functionalized with 9 M ethanolamine and treated with acetal-PEG-NHS (obtained from H. Gruber, Institute for Biophysics, University of Linz, Linz, Austria) at 2 mg/mL in a 6% (*v/v*) trimethylamine/chloroform solution. For integrin coupling, cantilever was treated with 100 µL 125 ng/mL integrin solution in HEPES for 1 h, at RT, to which 2 µL 1 M sodium cyanoborohydride was added subsequently. Force spectroscopy was performed with a NanoWizard BioAFM (JPK instruments AG, Berlin, Germany). The integrin-decorated cantilever was pressed to the substratum-bound laminin-111 for 1 s and retracted with a velocity of 300 nm/s. Retractions were repeated about 900 times. From every force–distance curve, the rupture force for the ultimate separation of cantilever from substratum was determined, to assure single molecular interactions. Force–distance curves showing unspecific cantilever–mica interactions, rupture distances below 20 nm or ambiguous last rupture events were rejected from evaluation.

### 2.11. Expression and Purification of Recombinant Redoxins

Transformed ClearColi^®^ BL21(DE3) cells (Lucigen, Middleton, WI, USA) with reduced endotoxicity were induced with 1 mM IPTG to produce His-tagged Trx1. After incubation overnight, at RT, bacteria were lysed with 20 mg lysozyme and DNAseI, and by sonification. From the lysate, Trx1 was purified with immobilized metal affinity chromatography columns (GE Healthcare Life Science, Buckinghamshire, UK), checked for purity by SDS-PAGE and tested to be endotoxin-free with the HEK-Blue™ LPS Detection Kit2 (Invitrogen). For cell experiments, recombinant proteins were rebuffered into TBS, reduced with 10 mM DTT for 30 min, at RT, desalted with Zeba Spin columns (Thermo Fisher Scientific), spectrometrically quantified and applied at 10 µg/mL.

### 2.12. Intermediate Trapping of Trx1 to Integrins

The trapping Trx1 C35S mutant [36] was expressed and purified as His-Tag fusion protein [37]. It was immobilized and reduced with 10 mM DTT on a His-Trap column, onto which 10–20 mg proteins of HT1080 cell lysate was loaded. Bound Trx1-substrates were eluted with 10 mM DTT and analyzed by immunoblot, with an integrin β1 antiserum [11], and by mass spectrometry. For the latter, tryptic fragments of trapped proteins were identified with a QExactive plus mass spectrometer [38]. Sequences were searched with MaxQuant (version 1.6.6.0, Max Planck Institute of Biochemistry) in a Homo sapiens reference proteome set (UniProtKB) supplemented with an entry for mouse integrin α7.

### 2.13. Impedance-Based Measurement of Cell Adhesion and Migration

For adhesion studies, E-plates (ACEA Biosciences, San Diego, CA, USA) were coated with 2 µg/mL laminin-111 in PBS, pH 7.4, overnight. After washing with PBS and blocking with 0.1% BSA in PBS, each well was filled with 50 µL Tyrode’s solution (140 mM NaCl, 5 mM KCl, 0.5 mM MgCl_2_, 1.8 mM CaCl_2_, 5 mM HEPES, 5 mM Glucose, pH adjusted to 7.4 with NaOH), with or without additional supplements (GoH3, H_2_O_2_). The cells were harvested, washed and resuspended at 1 × 10^6^ mL^-1^. Then, 50 µL thereof was added to each well. For migration assays, the bottom filter face of CIM-plates (ACEA Biosciences) was coated with 2 µg/mL laminin-111 solution in PBS, pH 7.4, overnight. The bottom compartment of the CIM plate was filled with 165 µL Tyrode’s solution, including 1% FCS as chemotactic stimulus. Then, 5 × 10^4^ cells/well in Tyrode buffer were added to the top compartment. After that, 10 µg/mL Trx1 or 10 µM H_2_O_2_ was added to both compartments. The experiments were performed at 37 °C, with 5% CO_2_. Cell adhesion and spreading in E-plates and cell migration in CIM-plates were recorded as the change of electric impedance (equivalent to “cell index”) by the xCELLigence^®^-system and the RTCA software 1.2 (ACEA Biosciences), at 5 min intervals, and corrected for the background values, to obtain Δ cell index values. Migration rate was determined as slope of Δcell index changes over time. Relative ΔΔ cell index values for each time point were obtained from the differences of Δcell index values of treated and nontreated samples, which were normalized to the Δcell index value of the nontreated sample.

### 2.14. Negative Staining and Electron Microscopy of Recombinant Soluble α7β1 Integrin

The conformation of integrin molecules was analyzed by negative staining electron microscopy [39]. Integrin samples (typically 25 µL) were treated with 10 µM H_2_O_2_ or 10 µg/mL thioredoxin-1, respectively, for 1 h at 37 °C. Carbon-coated 400 mesh copper grids were rendered hydrophilic by glow discharge at low pressure in air. Then, 5 mL specimen samples were adsorbed onto the hydrophilic grids for 1 min. After washing with two drops of water, the samples were negatively stained with two drops of 0.75% uranyl-formate. Specimens were examined, using a Philips/FEI CM 100 electron microscope, operated at an accelerating voltage of 80 kV; images were recorded with an OSIS Veleta side-mounted digital slow scan 2 k × 2 k CCD camera system, using Digital Micrograph^TM^ software (version 3.4, Gatan Inc., Pleasamton, CA, USA).

## 3. Results

### 3.1. Cysteines of the Integrin α7 Subunit Calf-2 Domain Are Structurally Important

**Figure 1:**
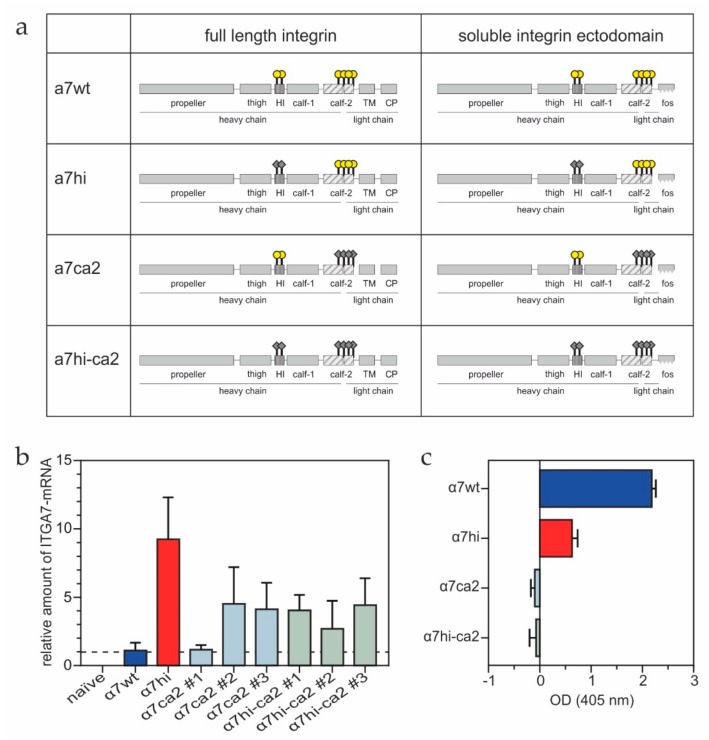
Schematic overview of integrin α7 constructs (**a**) and quantification of their transcription (**b**) and translation (**c**) in transfected HT1080 cells. (**a**) Redoxmodifiable cysteines of the integrin α7, located within the hinge region (C604, C610) and calf-2 domain (C862, C916, C923, C928) (yellow circles), were replaced by alanine residues (gray diamonds). HI, hinge (knee) domain; TM, transmembrane domain; CP, cytoplasmic domain; fos, heterodimerizing fos-zipper motif. (**b**) The qRT-PCR analysis of transfected integrin α7 constructs in HT1080 cells. To obtain relative values, the mRNA amounts of the α7 mutants were normalized to the α7wt value (dotted line). Means ± SD are shown for three independent experiments. Numbers (#1, #2 and #3) for α7ca2 and α7hi-ca2 indicate three independently transfected cell populations. (**c**) Sandwich-ELISA of whole HT1080 cell lysates (protein concentration: 1 mg/mL) performed with anti-integrin α7 mAb 3C12 and rabbit anti-integrin β1 serum as capturing and detecting antibody.

To mechanistically study the six oxidizable cysteine residues within the integrin α7 subunit [22], we mutated (i) the two cysteines within the hinge region, (ii) the four cysteines within the calf-2 domain and (iii) all six cysteines into alanine residues, thereby obtaining the mutants, α7hi, α7ca2 and α7hi-ca2, respectively (Figure 1a). After transfection of the human fibrosarcoma HT1080 cells with these full-length integrin-encoding cDNAs (Figure 1a, left column), the highest amount of transcribed mRNA was detected in α7hi-transfectants, as compared to the low transcription level of α7 wild type (α7wt) (Figure 1b). Three independent transfections with α7ca2 and α7hi-ca2 constructs (#1–3 in Figure 1b) yielded transcription of the respective cDNAs, yet none of them resulted in protein expression. Only the β1 integrin heterodimers with α7wt and α7hi were detected in the cell lysate (Figure 1c).

**Figure 2:**
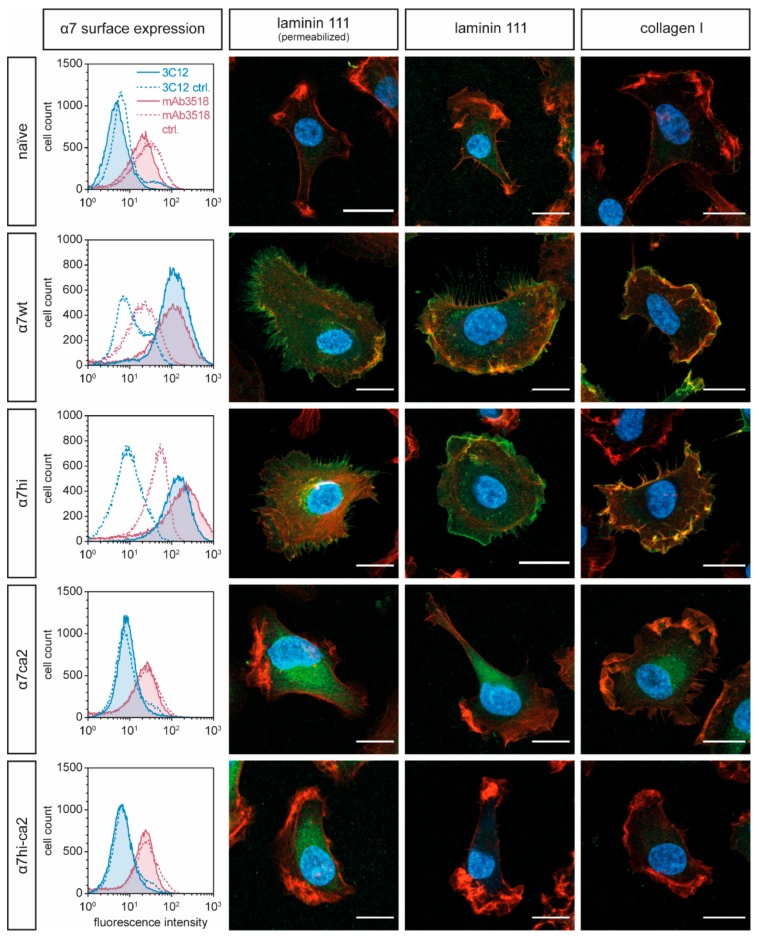
Subunits α7wt and the α7hi mutant, but not the α7ca2 and α7hi-ca2 mutants, are expressed as heterodimeric integrin on the surface of transfected HT1080 cells and induce morphological changes of cells after adhesion to laminin-111 and collagen-I. First column: surface exposure of integrin α7β1 wild type and mutants on transfected HT1080 cells was quantified by flow cytometry independently with two monoclonal antibodies, 3C12 and mAb3518 (clone #334908). Second to fourth columns: immunofluorescence of adherent transfectants on laminin-111 and collagen-I, either with (second column) or without (third and fourth column) permeabilization, and stained with an anti-integrin α7 antibody 3C12 (green), with phalloidin (red) and Hoechst dye (blue). Scale bars = 20 µm. Representative pictures of at least two independent staining experiments with at least 10 cells per condition are shown.

Surface expression of α7β1 integrin wild-type and mutants was quantified by flow cytometry, using two distinct mAbs, clones 3C12 and mAb3518. Only α7wt and α7hi heterodimers were exposed on the cell surface, but not the α7ca2- or α7hi-ca2 subunit (Figure 2, left column). To prove whether the latter two are aberrantly folded and hence retained along the secretory pathway, immunofluorescence microscopy of HT1080 adherent to laminin-111 was performed. Integrin β1 heterodimers containing either α7wt or α7hi were exposed on the cell surface, but none of the α7ca2 or α7hi-ca2 (Figure 2). Permeabilization of cells only slightly, if at all, increased the intracellular staining signal of the α7ca2 and α7hi-ca2 (Figure 2, second column). These data suggested that the mutant α7 integrins with a cysteine-free calf-2-domain were degraded intracellularly after translation. Therefore, the four cysteine residues within the calf-2 domain have a structural but not a regulatory role and are indispensable for proper folding of α7β1 integrin.

### 3.2. The Cysteine Pair within the α7 Integrin Hinge Region Determines the Morphology of Transfected HT1080 Cells

The only expressed mutant, α7hi, was further analyzed. To this end, HT1080 cells transfected with α7wt- or α7hi-encoding cDNA constructs were morphologically compared on different ECM substrates, laminin-111 and collagen-I. On laminin-111, a ligand for α7β1 integrin, HT1080 cells expressing α7wt showed large lamellipodia and spread fully, whereas α7hi transfectants spread less (Figure 2). In both transfectants, the highest density of surface-exposed integrin α7β1 was observed close to the cortical actin network at the cell perimeter. On collagen I, an integrin α7β1-independent substrate, both α7wt and α7hi transfectants spread indistinguishably from each other, and integrins α7wtβ1 and α7hiβ1 were found in actin-containing filopodia and membrane ruffles above the cell soma.

**Figure 3:**
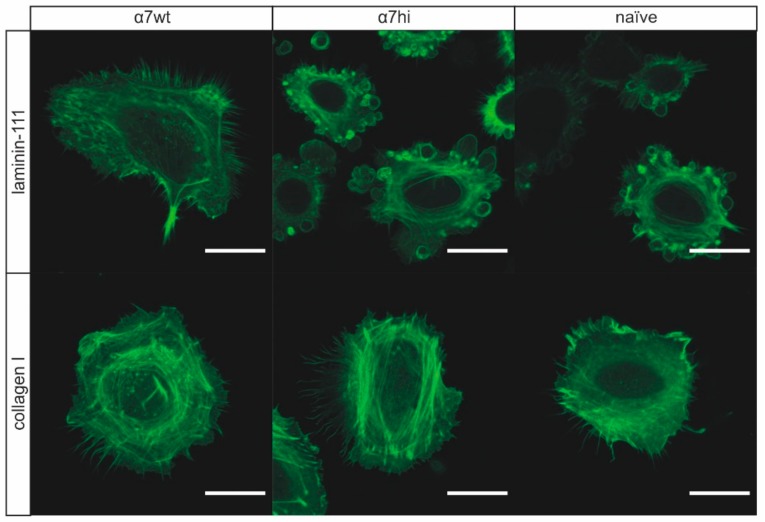
Cell morphology of HT1080 cells expressing α7wt and α7hi in life cell microscopy. Integrin α7hi, but not α7wt, induces bleb-like membrane protrusions in transfected HT1080 cells when plated on laminin-111, but not on collagen I. LifeAct-GFP-transduced HT1080 cells were monitored by life cell microscopy on µ-slides coated with laminin-111 or rat tail collagen I (ColI). Images were taken with confocal microscopy with 40× oil objective at 37 °C. Scale bar = 20 µm. Maximum projection of z-stacks was done with FiJi. Representative images from at least 30 cells for each condition are shown.

Next, we compared LifeAct-GFP-transfected HT1080 cells bearing α7wt and α7hi in live-cell imaging, thereby preventing fixation artefacts (Figure 3). On collagen-I, HT1080 cells spread fully and formed lamellipodia and a fringe of filopodia, irrespective of the α7 subunit. In contrast, on laminin-111, only HT1080 cells expressing integrin α7wt developed lamellipodia decorated with numerous filopodia, whereas the α7hi-transfected cells formed bleb-like cell membrane protrusions. Due to their fragility, these blebs were not detected in cells after fixation and mounting underneath a cover slip (Figure 2). Moreover, non-transfected HT1080 cells formed such blebs, albeit of smaller size (Figure 3). These different cell protrusions were likely caused by different adhesion strengths of two α7β1 integrin forms.

### 3.3. α7hi Supports Cell Migration Less Than Integrin α7wt, Despite a Similar Adhesion Reaction

**Figure 4:**
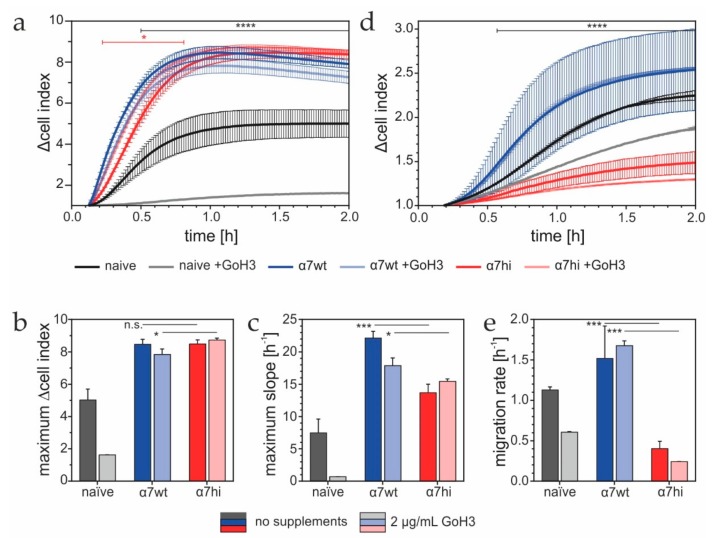
Adhesion and migration of HT1080 cells expressing integrin α7wt and α7hi. (**a**) Adhesion to laminin-111of naïve and transfected HT1080 cells in an E-plate of the xCELLigence DP device. Cells expressing α7wt or α7hi adhered faster and with a higher saturation signal than the naïve HT1080 cells. Adhesion via α6β1 integrin was challenged by adding the antibody GoH3. (**b**) Maximum adhesion signals and (**c**) maximum slope, indicating the maximum adhesion rate, were compared for at least three independent experiments. Means ± SD are shown. (**d**) Haptotactic migration toward laminin-111 of HT1080 cells was recorded in a CIM plate of the xCELLigence DP device. Cells expressing α7hi moved much slower than α7wt transfectants and naïve cells. (**e**) The migration rates were determined as change of Δcell index values over time, between 30 and 60 min. Experiments were performed in Tyrode’s solution. Means ± SD of a quadruplet determination of one out of three experiments are shown. The data in (**a**) and (**d**) were compared for the effect of GoH3 by two-way-ANOVA with multiple comparison correction via the Holm–Sidak method. Time periods with significant differences are marked in the corresponding color of the cells. As only the naïve HT1080 showed significant changes upon GoH3 treatment after 1 h, the comparison bars for comparing the GoH3-free vs. GoH3-treated samples are omitted in (**b**), (**c**) and (**e**). In the same plots, comparison bars are only shown for the comparison of α7wt vs. α7hi transfectants and are based on pairwise comparisons with Student’s *t*-test. Significance levels: * *p* ≤ 0.05; ***. *p* ≤ 0.005; ****, *p* ≤ 0.001.

Adhesion and migration of α7wt and α7hi transfectants to laminin-111 were assessed by impedance-based measurements in real time. Since HT1080 cells also expressed integrin α6β1, another receptor for laminin-111, we used the integrin α6-blocking antibody GoH3, to study the role of α7β1 exclusively. The adhesion of non-transfected HT1080 cells was abolished almost completely, indicating that α6β1 integrin is the predominant adhesion receptor for laminin-111 on non-transfected HT1080 cells (Figure 4a). Only for the naïve HT1080 cells, the maximum Δcell index (Figure 4b) and the maximum slope (Figure 4c) showed significant differences upon GoH3 treatment. In contrast, irrespective of GoH3, cells transfected with α7wt or α7hi adhered with similar kinetics (Figure 4a) and reached similar maximum adhesion signals (Figure 4b). This indicated that integrin α7β1 had become the principal adhesion receptor for laminin-111 in transfected cells. Despite their different morphologies (Figure 3), both transfectants similarly adhered with higher adhesion signals than naive HT1080 cells (Figure 4b). The time-dependent increase of impedance values, measured as the maximum slope of the adhesion kinetics in the initial phase, was significantly lower for α7hi- than α7wt-transfected cells, presumably due to the less intense cell contacts with the laminin-111-coated electrodes via the bleb-like cell protrusions than via lamellipodia (Figure 4c).

In contrast to adhesion, HT1080 cells expressing integrin α7hi migrated toward laminin-111 significantly slower than the α7wt-transfected cells, and even slower than the non-transfected controls, irrespective of the presence of GoH3 (Figure 4d,e). The irrelevance of GoH3 demonstrated that, also for migration toward laminin-111, integrin α7β1 plays the principal role in transfected cells. Moreover, the lack of the cysteine pair within the α7 hinge decreased cell migration, but not adhesion, to laminin-111.

### 3.4. Oxidation of the Cysteine Pair in the Integrin α7 Hinge Results in H_2_O_2_-Promoted Migration of α7wt-, but Not of α7hi-Expressing Cells

**Figure 5:**
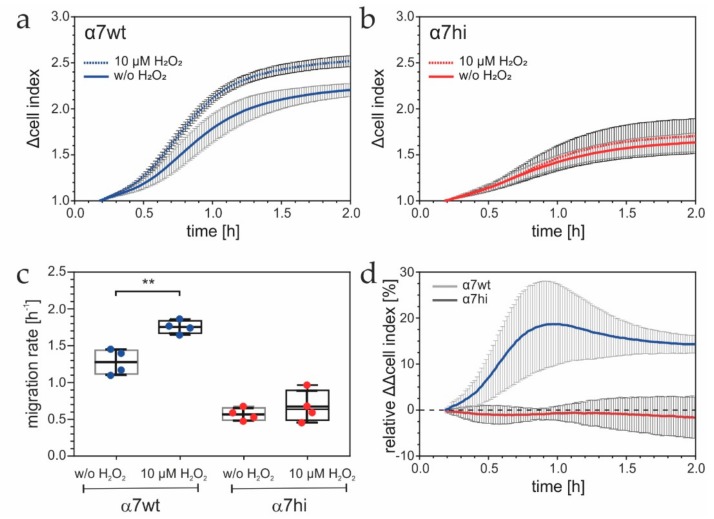
Effect of H_2_O_2_ on integrin α7-dependent migration. Migration on laminin-111 of HT1080 cells expressing integrin α7wt (**a**) or α7hi (**b**) was monitored on CIM-plates in an xCELLigence DP device, in the absence and presence of 10 µM H_2_O_2_. The experiments were carried out in Tyrode’s solution and in the presence of 2 µg/mL GoH3. Means ± SD. of Δcell index values, measured in 5 min intervals, from at least four independent measurements are shown. The Holm-Sidak method revealed a significant difference (*p* < 0.05) for α7wt-, but not α7hi-transfectants, after the first 60 min of H_2_O_2_ treatment. (**c**) The average migration rates between 30 and 60 min after migration start are shown from one of three experiments with quadruplet determination with means ± SD. Significance level: ** *p* ≤ 0.01. (**d**) Relative ΔΔcell index values were calculated at each time point for HT1080 cells expressing α7wt or α7hi to compare H_2_O_2_-treated and nontreated samples. Means ± SD are shown (*n* = 4 for α7wt and *n* = 6 for α7hi).

To test the hypothesis that the pair of cysteines within the hinge motif can be oxidized and consequently influences α7β1 integrin and its cell functions, chemohaptotactic migration of α7wt- and α7hi-transfected HT1080 cells toward laminin-111 was compared in the absence vs. presence of 10 µM H_2_O_2_ (Figure 5). To avoid nonspecific decomposition of H_2_O_2_, Tyrode buffer without any redox-relevant components was used. The α7wt transfectants showed higher Δcell index values in the migration chamber, in response to H_2_O_2_, than nontreated control cells did (Figure 5a), whereas α7hi transfectants were not stimulated by H_2_O_2_ (Figure 5b). Thus, the migration rates, which were already significantly higher for α7wt than for α7hi transfectants in the absence of H_2_O_2_, changed differently in its presence (Figure 5c). These H_2_O_2_-induced differences became even clearer when they were normalized to the nontreated sample (Figure 5d). These relative ΔΔcell index values of α7hi-transfected cells did not significantly differ from zero over time, showing that these cells did not respond to H_2_O_2_, whereas they rose significantly for α7wt transfectants and persisted over at least two hours (Figure 5d). These data proved that the oxidation of the cysteine pair in the α7 hinge promoted α7β1 integrin-mediated cell migration.

### 3.5. The Wild-Type α7β1 Integrin Occurs in a High Activity-Conformation on the Cell Surface at a Higher Frequency Than the α7hi-Mutant

**Figure 6:**
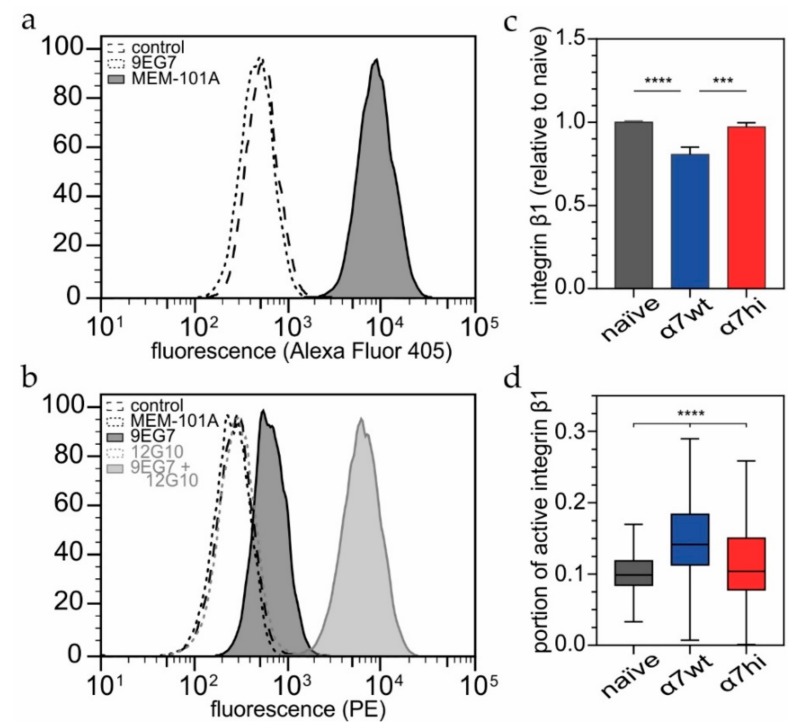
Surface-exposed α7β1 integrin containing the α7wt, but not the α7hi mutant, preferentially takes the active/extended conformation with an accessible 9EG7 epitope. (**a**,**b**) Representative flow cytometric histograms, such as the ones shown here for α7wt transfected HT1080 cells, reveal (**a**) all β1 integrin heterodimers, irrespective of their conformational state, after staining with anti-β1 mAb MEM-101A and Alexa Fluor 405-conjugated secondary antibodies. No cross reaction was observed for 9EG7 as primary antibody. (**b**) Integrin β1 molecules in an extended conformation stained with biotinylated 9EG7 and secondarily with phycoerythrin (PE)-conjugated NeutrAvidin. No cross reaction was detected for MEM-101A or 12G10 as primary antibodies. To prove 9EG7 functionality, β1 activating antibody 12G10 was added along with 9EG7, representing the maximum signal reachable with 9EG7. (**c**) All surface-exposed β1-integrins were detected with MEM-101A antibody in a conformation-independent manner. The quantity of β1-integrins was lower in HT1080 cells transfected with the α7wt construct. (**d**) 9EG7 detected the portion of active conformation of integrin β1 relative to the maximum reachable signal in presence of 12G10. Box plots indicate medians, 25th and 75th percentile, and Tukey whiskers. In total, 12,720, 9117 and 8349 events were evaluated for naïve, α7wt and α7hi, respectively. ANOVA with Tukey’s multiple-comparisons test was performed. Significance levels are indicated by asterisks (*** *p* ≤ 0.005; **** *p* ≤ 0.001).

We hypothesized that the molecular basis of increased cell migration under oxidizing conditions correlates with an activation and a conformational change of the integrin ectodomain. As the epitope of the mAb 9EG7 within the integrin β1 subunit is accessible only in the extended conformation, we employed this antibody to assess the portion of extended integrin conformation within the entire cell surface population of β1 integrins. The latter was determined with the anti-integrin β1 antibody MEM-101A that recognizes its epitope conformation independently. Being antibodies from different species, 9EG7 and MEM-101A were simultaneously used in flow cytometry (Figure 6a). As positive control, the β1 integrin-activating antibody, 12G10, revealed the total fraction of activatable β1 integrins (Figure 6b). The flow cytometric MEM-101A signal showed that the β1 integrin expression in HT1080 cells transfected with the α7hi construct was equally high as in non-transfected cells. Only the α7wt-transfectants exposed less β1 integrin on their surface (Figure 6c). Nevertheless, the α7wt-transfected cells revealed a significantly higher portion of accessible 9EG7 epitopes than the non-transfected cells and α7hi transfectants (Figure 6d). The little, yet significantly increased portion of 9EG7-accessible β1 integrins is especially striking as this test detected not only the extended conformation of active α7β1 integrin, but of all β1 integrins. Therefore, we concluded, that a higher portion of the wild type α7β1 integrin molecules took the extended conformation and likely possess higher ligand binding activity as compared to the α7hiβ1 integrin molecules, which remained in the less-active bent conformation on the cell surface.

### 3.6. Oxidation of the Cysteine Pair Within the Hinge Region of Integrin α7β1 Increases Binding Activity and Force to Laminin-111

**Figure 7:**
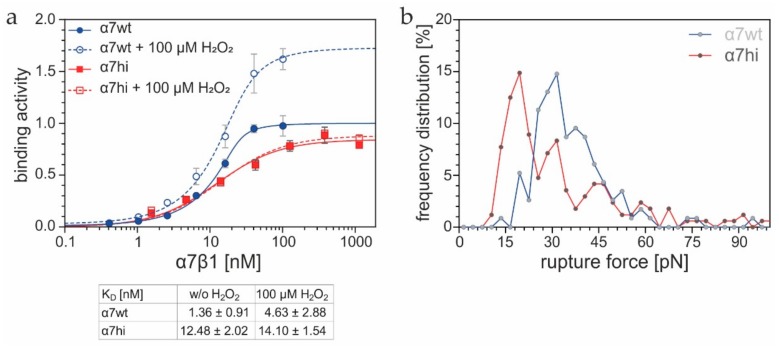
Ligand-binding activity and binding force of α7 integrins toward laminin-111. (**a**) Soluble integrins, α7wtβ1 and α7hiβ1, pretreated with or without 100 µM H_2_O_2_, were added to immobilized laminin-111. Means (±SEM) of triplicates of a representative experiment are shown. At least six titration curves for each condition were fitted according to [35], to obtain K_D_ values. (**b**) AFM-based force measurements on single molecule interactions between integrins, α7wtβ1 and α7hiβ1, and laminin-111. After the cantilever with immobilized integrin was approached to the laminin-111 coated surface, it was retracted with a velocity of 300 nm/s. Force curves for α7wt and α7hi were measured two times, with three loading rates. Rupture force of the last contact was evaluated for 331 and 168 significant force–distance curves for α7wt and α7hi, respectively. For the histogram, the results were binned with a force width of 3 pN.

To analyze the molecular mechanism of the thiol-based redox regulation within the integrin α7 hinge, we recombinantly produced the corresponding soluble α7β1 integrin ectodomains (Figure 1, right column). Both α7wtβ1 and α7hiβ1 integrins interacted with immobilized laminin-111 in the protein–chemical binding ELISA (Figure 7a). However, the binding signals of soluble α7wtβ1 were consistently higher than the ones of α7hiβ1, presumably in correlation with the more active conformation. Moreover, α7hiβ1 had a lower affinity to laminin-111 than the wild-type form. When laminin-111 binding was challenged with 100 µM H_2_O_2_ (Figure 7a), the dissociation constant of α7wtβ1, but not of α7hiβ1, increased slightly. More strikingly, the saturation binding signals of α7wtβ1, but not of α7hiβ1, rose remarkably by more than 72% upon H_2_O_2_ treatment, indicating an increase of integrin molecules with the higher activity conformation. Thus, the cysteine pair within the integrin α7 hinge is sufficient to redox-regulate ligand-binding activity. Moreover, the dramatic changes of the saturation-binding signals of α7wtβ1 integrin, in combination with the subtle change of K_D_-values upon H_2_O_2_ treatment, suggested that the oxidation of the cysteine pair within the α7 hinge rather acts allosterically instead of affecting the laminin binding site.

To determine molecular forces, rupture forces were measured upon retraction of the integrin-coated cantilever from a laminin-111-coated surface. In the histogram of these yield forces, both integrins showed several peaks of different heights, potentially representing interactions between one, two and more integrin–ligand pairs (Figure 7b). Consequently, the highest peak at lowest yield force represented the single molecule interactions. It showed a similar frequency for both integrin forms. However, most α7wtβ1 integrin molecules, when bound to one laminin-111 molecule, withstood forces of about 30 pN, whereas the highest portion α7hiβ1 integrin molecules released its bound laminin-111 molecule at lower forces, of about 16 pN.

### 3.7. The Integrin Ectodomain Is a Substrate for Thioredoxin-1, Which Reduces the Integrin α7wt Thiol-Switch

**Figure 8:**
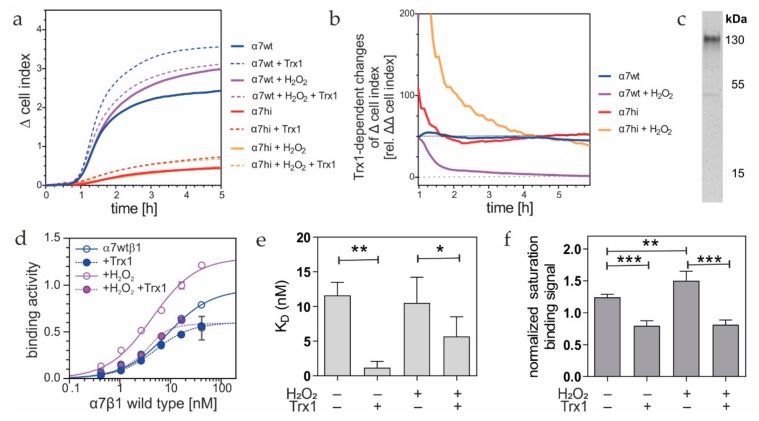
Extracellular thioredoxin-1 (Trx1) reduces cell migration and binding to laminin-111, mediated by α7wtβ1, but not by α7hiβ1 integrin. (**a**) Migration of HT1080 cells toward laminin-111. HT1080 cells expressing integrin α7wt (blue and purple lines) or α7hi (red and orange lines) were pretreated without and with 10 µM H_2_O_2_ for 30 min and washed. Migration was monitored on CIM-plates in the xCELLigence DP device, in the absence (full line) and presence (dashed line) of 10 µg/mL reduced Trx1 in Tyrode’s solution. Means of triplicate Δcell index values from at least three independent measurements are shown. The variation coefficients varied around 10–20% on average. (**b**) For every time point, relative ΔΔcell index values were calculated from the data in (**a**), revealing the Trx1-dependence of the measured values. Means of triplicate values are shown for one out of three representative experiments. (**c**) Immunoblot detection of β1 integrins in the eluate of a Trx1-trap mutant affinity column. The Trx1 trap mutant with its Cys-X-X-Ser active site retained Trx1-bound proteins from a cell lysate of α7wt-transfected HT1080 cells. In parallel, the eluate contained inter alia the integrin α7 subunit, as proven by mass spectrometry (Appendix A, Figure A2). (**d**) Titration of laminin-111 with soluble α7wtβ1 integrin, pretreated without and with 100 µM H_2_O_2_ (blue and purple lines). After removal of H_2_O_2_, binding was tested in the presence (dashed lines) and absence (full line) of 10 µg/mL Trx1, including a subsequent NEM-treatment, to prevent thiol oxidation. One triplicate set of two independent titration experiments are shown. (**e**) K_D_-values, and (**f**) saturation binding signals of six independent titration curves (as shown in (d)) were evaluated, and their means ± SD are shown. Significance levels are indicated by asterisks: * *p* ≤ 0.05; ** *p* ≤ 0.01; *** *p* ≤ 0.005.

**Figure A1:**
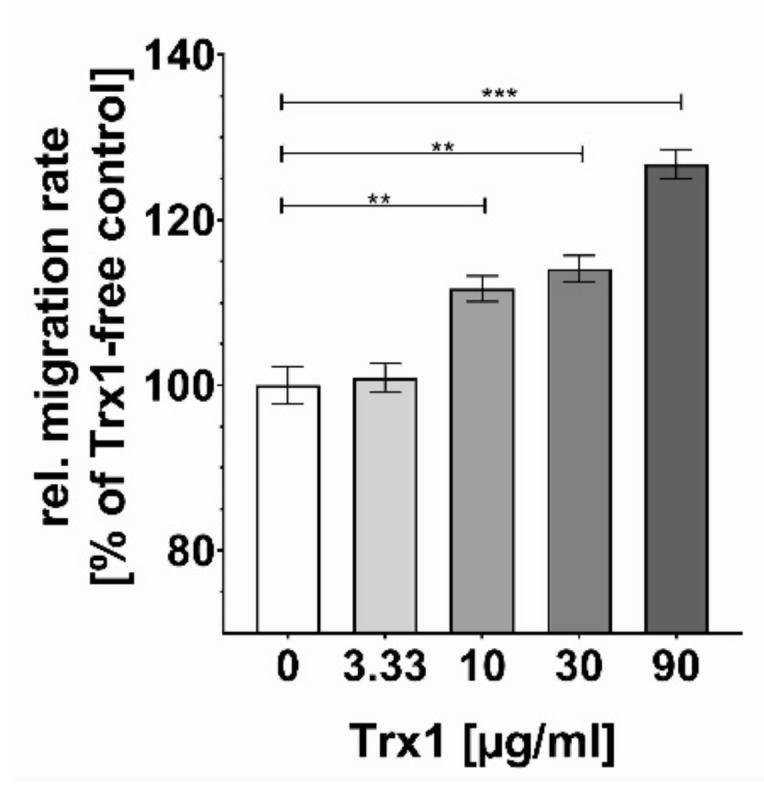
In a dose-dependent manner, reduced Trx1 promotes chemohaptotactic migration of non-transfected HT1080 cells toward poly-l-lysine in an integrin-independent manner. The bottom face of a CIM-plate was coated with 0.01 poly-l-lysine at 4 °C overnight. A group of 5 × 10^4^/well non-transfected HT1080 cells was allowed to migrate in the presence of different concentrations of reduced Trx1. The Δcell index values were monitored. From them, relative ΔΔcell index values were calculated. Means ± SD of triplicates from a representative experiment out of three experiments are shown. Significance levels: ** *p* ≤ 0.01; *** *p* ≤ 0.005

These cellular and molecular findings suggested that the hinge-located pair of cysteines constitutes a thiol-switch that, depending on its redox state, alters the conformation and ligand-binding activity of the integrin. A thiol-switch must be reversible to be part of a redox signaling circuit. Since this putative hinge thiol-switch is located in the integrin ectodomain, it should be accessible to and cleavable by an extracellular reducing agent, such as the oxidoreductase Trx1. To test this hypothesis, α7wtβ1- and α7hiβ1-bearing HT1080 cells, either without or with prior treatment with H_2_O_2_, were allowed to migrate toward laminin-111, in the presence or absence of reduced Trx1 (Figure 8a). To reveal the effect of reduced Trx1 on cell migration, the relative ΔΔcell index values were determined for each time point. Three of the four pretreatment conditions, i.e., (i) α7wt without H_2_O_2_ pretreatment, (ii) α7hi with H_2_O_2_ pretreatment and (iii) α7hi without H_2_O_2_ pretreatment, showed a clear Trx1-dependent increase in relative ΔΔcell index values of at least 50% in the first 5 h of the experiment (Figure 8b). We hypothesized that this increase is a pleiotropic promigratory effect of Trx1 on cells that is not related to integrins. In fact, Trx1 dose-dependently supported cell migration on integrin-independent substrates, e.g., poly-l-lysine (Appendix A, Figure A1). In contrast to the three other pretreatment conditions, only in the fourth condition, the α7wt transfectants with H_2_O_2_ pretreatment did not migrate toward laminin-111, in the presence of reduced Trx1 with a similarly proportional increase (Figure 8a). They showed remarkably lower Trx1-dependent relative ΔΔcell index values, which approached zero values (Figure 8b, purple line). This suggests that reduced Trx1 attenuated the activated integrin α7wtβ1 with its previously H_2_O_2_-oxidized thiol-switch by reducing it and reverting the integrin into its lower activity conformation. Our data indicate that the hinge-located pair of cysteines constitutes a regulatory thiol-switch, which, as a dithiol, is activated by H_2_O_2_ and, as a disulfide bridge, is attenuated byTrx1, respectively. Neither oxidative nor reductive conditions affected the a7hi mutant.

**Figure A2:**
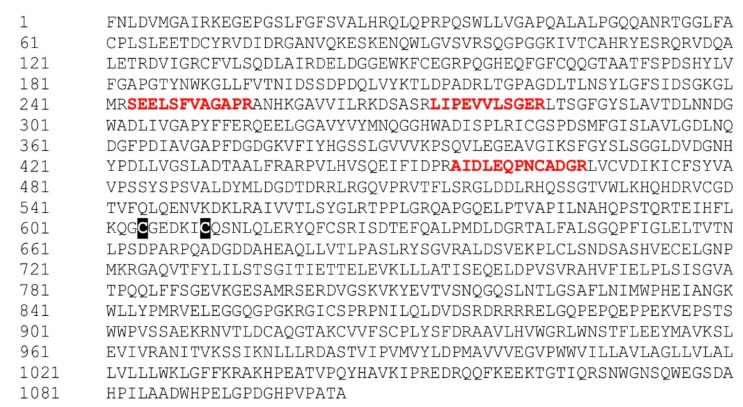
Mass-spectrometric analysis of proteins trapped by Trx1-trap mutant. The Cys-X-X-Ser mutant of Trx1 was applied in an intermediate trapping approach to trap Trx1 substrates on α7wt-HT1080 cells. The trapped proteins were analyzed after tryptic digestion, using liquid chromatography coupled with mass spectrometry. Among the trapped proteins, integrin α7 was identified with a MaxQuant score of 22.9 with three individual peptides (marked in red). All three peptides were identified at a 2+ charge state, with individual MaxQuant scores of 76.5 (AIDLEQPNCADGR, PEP 1.1 E-7), 44.9 (LIPEVVLSGER, PEP 0.0027) and 36.7 (SEELSFVAGAPR, PEP 0.0055). The two cysteines of the hinge motif are highlighted with black boxes.

At the molecular level, we aimed to prove a direct interaction of Trx1 and α7β1 integrin. The intermediate trapping approach employs a Trx1 trapping mutant with one mutated cysteine in the active site. It detects substrates that undergo Trx1-catalyzed dithiol–disulfide exchange reactions. This Trx1-trapping mutant was immobilized as a bait on an affinity column. The eluted proteins stained positive for β1 integrin in the immunoblot (Figure 8c), and mass spectrometric analysis identified the integrin α7 subunit among the Trx1-trapped proteins (Appendix A, Figure A2). Hence, Trx1 interacted with α7β1 integrin, which thus proved to be a potential redox substrate of Trx1.

To substantiate this hypothesis further, we recapitulated the Trx1-mediated reduction of α7wtβ1 integrin after H_2_O_2_ oxidation at the molecular level in an in vitro assay. Although prior oxidation of α7wtβ1 integrin increased the binding signals to laminin-111, both non-H_2_O_2_-treated and H_2_O_2_-oxidized α7wtβ1 integrin approached similar low binding values in the presence of Trx1 (Figure 8d). Trx1 reduced the K_D_-values of α7wtβ1 integrin to laminin-111, albeit not by orders of magnitude (Figure 8e). Even more significantly, Trx1 decreased the saturation binding signals (Figure 8f), in line with the lower portion of molecules in the extended conformation of the integrin population.

**Figure 9:**
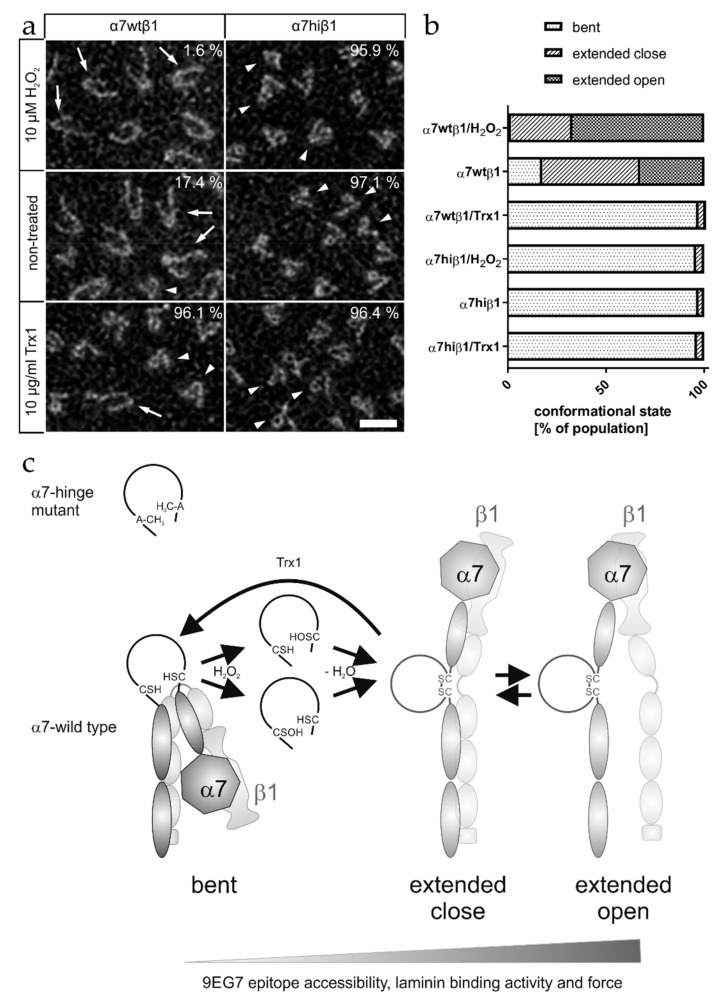
Electron microscopy of recombinant soluble α7β1 integrin after negative staining. (**a**) The electron micrographs show representative fields of α7wtβ1 (left column) and α7hiβ1 (right column) molecules (scale bar: 20 nm). The tadpole-like complexes with one or two tails exhibit both bent (arrowheads) and extended (arrows) integrin forms. The percentage of molecules in different conformations was determined from at least 500 molecules under each condition (top row: no additives; middle row: 10 µM H_2_O_2_; bottom row: 10 µg/mL Trx1). The percentage of bent conformation is printed in each image. (**b**) Quantification of conformations of integrin molecules from electromicrographs, such as in (**a**), including a differentiation of the extended conformation into an extended close and extended open conformation. (**c**) Scheme of the thiol-switch mediated conformational changes of α7β1 integrin. The integrin hinge region is indicated as the loop in the central pivot region of the integrin molecule. Rotation around the hinge ensures the conformational equilibrium between bent and extended form. The pair of cysteines within the α7 hinge in the bent conformation is oxidized with H_2_O_2_ to mono-cysteine-sulfenic acid derivatives, which form a disulfide bond. This stabilizes the extended conformations. The disulfide-bonded form of the thiol-switch can be reduced by extracellular Trx1. This is accompanied with transition into the bent conformation. Mutation of both cysteines for alanines (upper left panel) represents the loop structure of the less active α7hi. The thiol-switch-dependent conformational changes correlate with changes in ligand binding, in 9EG7 epitope recognition and in cell migration.

To prove their conformations at the molecular level, the integrins, α7wtβ1 and α7hiβ1, were visualized by transmission electron microscopy after negative staining (Figure 9a). Only 17.4% of the α7wtβ1 integrins took a bent conformation, which, in contrast, was found for 97.1% of the α7hiβ1 molecules. Treatment of the latter with neither H_2_O_2_ nor Trx1 changed the conformational equilibrium (Figure 9a, right column). In contrast, the percentage of bent conformations within the α7wtβ1 decreased from 17.4% to 1.6% after oxidation with H_2_O_2_, whereas it drastically increased to 96.1% after Trx treatment (Figure 9a, left column). Moreover, further differentiation of integrin population of the extended conformation [5] revealed that treatment with H_2_O_2_ converted α7wtβ1 integrin molecules not only into the extended close, but also into the extended open conformation (Figure 9b). Trx1 had a converse effect. In contrast, α7hiβ1 integrin did not change their conformation at all and remained in the bent conformation (Figure 9b).

These structural data showed that the hinge-located cysteine pair within integrin α7β1 forms a thiol-switch, which dominantly governs the conformation of the integrin. It is redox-regulated by extracellular H_2_O_2_ and Trx1. The former causes formation of a disulfide bridge and conversion of the integrin into the extended open conformation with high ligand-binding activity, whereas the latter conversely reduces the disulfide bridge and converts the integrin in a bent conformation with low ligand-binding activity. This redox-dependent conformational conversion results in redox-dependent migration of α7β1 integrin-expressing cells toward laminin-111.

## 4. Discussion

Integrins possess numerous cysteine residues within their ectodomains. At least 56 cysteines are located within the integrin β subunit, and integrin α chains encompass between 19 and 35 cysteine residues [19,40]. The formation of disulfide bridges is largely conserved among the different integrin α and β subunits. However, the controversially reported patterns of some disulfide bridges might not only be due to differences between their crystallographic and protein–chemical mapping [41,42], but also due to different conformations of integrins. Previous observations showed that the patterns of disulfide bonds and free thiol groups of cysteine within their ectodomains may influence ligand binding of integrins [28]. They had been studied for the fibrinogen receptor αIIbβ3, for the collagen-binding integrin α2β1 on platelets [30,31,43] and for β2 integrins on neutrophils [44]. The disulfide bonds can be cleaved by reducing agents or even by photolysis with UV-C light [45]. Data published by us and others also demonstrated that H_2_O_2,_ at physiological concentrations of up to 100 µM forms disulfide bridges and activates β1 integrins, α7β1 and α4β1 [22,46]. Beyond those findings, here, we pinpoint one specific thiol-switch (Cys604-C610) and show its dominant role in binding activity and integrin conformation. In this study, we mutated six particular cysteine residues, which we had previously mapped to be oxidizable cysteines [22]. By comparing results from molecular and cellular studies, we not only verified the importance of the eminent cysteine pair, Cys604 and Cys610, within the α7 hinge, but also unraveled that this cysteine pair is fully sufficient to implement a redox-dependent change of conformation and ligand binding activity on α7β1 integrin. Hence, it is the dominant regulatory thiol-switch. Conspicuously, this cysteine pair is highly conserved in the integrin α subunits, implying the hypothesis that other integrins undergo a similar thiol-based redox regulation via this particular hinge-located thiol-switch.

Several other disulfide bonds within integrin ectodomains are in discussion to fulfill similar redox-regulatory roles, such as cysteine pairs within the β3 subunit located within the I-domain [27], within the EGF-domains of the stalk region [24,28,29,30], or between the PSI- and EGF1-domain as a long-range cysteine bond [31]. Within the integrin α subunits, cysteine bridges within the propeller domain and in the hinge region of integrin subunit α4 were delineated for their reducibility [24,25,26]. However, the integrin α4 subunit stands apart from other integrins because of its specific proteolytic processing likely within the thigh domain close to the hinge region and because of its easy dissociation into a heavy and light chain [47]. It is noteworthy that simultaneous mutation of all four redox-modifiable cysteine residues within the calf-2 domain in our study abolished stable expression of the respective α7 subunit due to their structural functions. Other examples of such structurally relevant disulfide bonds within other integrin subunits are of clinical relevance [48].

The biochemical binding tests revealed that replacement of the thiol-switch-forming cysteines for two alanine residues did not entirely abolish the binding affinity of α7β1 integrin for laminin-111. This is in line with the fact that the putative ligand-binding head domain, which consists of the α7 chain propeller domain and the β1 I-domain, is located in a distance from the hinge region. Hence, modification of the hinge region acts allosterically and the corresponding conformational change explains why the saturation signal, but not the affinity, of the hinge mutant changed remarkably in comparison to the wild type form. Thereafter, and in correlation with our electron microscopy-based conformational studies, the proportion of α7β1 integrin molecules in the extended open conformation with enhanced ligand binding activity is higher in the population of α7wt than α7hi mutant. This is also in line with the higher flow cytometric signal of the 9EG7 antibody in the cell-bound population of α7wtβ1 than of α7hiβ1, as the 9EG7 epitope located within the EGF-domains of the β1 subunits is accessible only in the extended conformation of β1 integrins [5,6,7]. Undergoing the common receptor recycling, integrins are taken up by cells especially in an active conformation [49]. This may explain the lower surface expression of β1 integrins in α7wt- than in α7hi-transfectant cells, as detected by the conformation-independent MEM-101A antibody.

The α7hiβ1 integrin molecule withstands lower retraction forces than the corresponding wild-type molecule in AFM measurements. In a previous study, we showed that molecular binding forces between the α7β1 wild-type integrin and its ligand invasion comply with a catch-bond model including two barriers [50,51]. Although measured at the same low loading rate, the yield forces of wild type and mutant integrin differed in their frequency distributions and minimum yield force. This is likely due to the different percentages of extended vs. bent conformation in the populations of integrin molecules, in line with the weaker interaction of α7hiβ1 vs. α7wtβ1 integrin with laminin-111. This lower binding activity of the α7β1 molecule lacking only the thiol-switch within the α7 hinge region contrasts with the published finding that a mutant of α4β7 integrin, in which cysteine pairs within the knee regions of both subunits were mutated, showed higher and constitutive activity [24]. This discrepancy might be caused by simultaneous modifications of pivot regions of both subunits or by the unique posttranslational proteolysis and subunit dissociation propensity of the α4β7 integrin [47].

Our molecular data were complemented with data measured at the cellular level. Expressed by HT1080 cells, the thiol-switch-deficient α7β1 mutant caused less spreading and induced a different cell morphology on laminin-111, as compared to the wild type form. The bleb-like cell protrusions of the α7hi transfectants indicate a weak cell adhesion to laminin-111, in accordance with its weaker molecular interaction with its ligand. This also explains the significantly weaker migration of the hinge mutant-transfected cells, as migration depends on repetitive attachment and detachment events. Our data are in agreement with the observation that poor integrin–matrix interactions reinforce an amoeboid migration mode, in which cells move by forming such bleb-like cell protrusions through the activity of their cortical actin network [52,53].

The molecular basis of redox-regulatory proteins can be a thiol-switch which may consist of a pair of cysteines, whose side chains’ reversibly change between a dithiol form and a disulfide configuration. The resulting conformational change within the integrin shifts the conformational equilibrium within the integrin population when oxidizing and reducing agents act specifically and alternately (Figure 9c). We conclude that H_2_O_2_ oxidizes the thiol groups of the two vicinal cysteine residues within the hinge loop via a mono-sulfenic acid derivative into a disulfide bridge. Only the wild-type α7β1 integrin, but not the α7hiβ1, can be oxidized within its hinge region to result in a disulfide bridge and an extended conformation. With its oxidized thiol-switch, the integrin binds to laminin-111 with stronger signals in the titration-ELISA, in molecular force measurements and in cell adhesion experiments. Noteworthy, the concentrations of H_2_O_2_ applied in protein–chemical experiments were one order of magnitude higher than the ones in cellular experiments. This difference is reasoned by different factors, such as the stoichiometric ratio between the integrin and H_2_O_2_ and sensitivity of the cells against H_2_O_2_. In protein–chemical assays, the molar concentration of isolated α7β1 integrin is higher than in cell experiments and thus requires higher molar concentrations of H_2_O_2_ to maintain comparable stoichiometric ratios. Various cells can produce cell-membrane-permeable H_2_O_2_ inter alia by the activity of the NADPH-oxidase-4 (NOX4) [54,55]. Previously, we showed that vascular smooth muscle cells transiently form H_2_O_2_ in a NOX4-dependent manner at spatially restricted sites [22]. These redox hot spots coincide with cell protrusions, where cells take the first contact with the extracellular matrix during the initial step of cell adhesion. They are transient, and H_2_O_2_ production ceases after cells have established an initial adhesive contact [22]. Here, we also demonstrated that extracellular Trx1 may reduce the thiol-switch within the integrin. It reduces the disulfide bond within the integrin α7 hinge region and converts the fully active integrin into the less active bent conformation (Figure 9b). This may lead to less firm cell attachment or even detachment.

In the Trx1 system, electrons are donated from NADPH to the flavo- and selenoprotein Trx reductase that reduces the oxidoreductase Trx1. Trx1 itself reduces disulfides and single cysteine modifications in several substrate proteins, e.g., nuclear factor κB and peroxiredoxins (reviewed in [32]). Both Trx1 and Trx reductase are reportedly secreted by various cells [56,57] and belong to the scarcely occurring and not yet well-characterized extracellular reducing agents [32]. Extracellular Trx1 redox regulates immune mediators and signal-transmitting ion channels [58], such as macrophage migration inhibitory factor (MIF) [59], tumor necrosis factor receptor superfamily member 8/CD30 [60] and the transient receptor potential channel 5 (TRPC5) [59]. Our study adds a new substrate, integrin α7β1, to the list of redox-regulated Trx1 substrates and most likely also other integrins which are crucial for the adhesive and migratory interaction and signal transduction between cells and the ECM.

In summary, extracellular Trx1 and H_2_O_2_ constitute parts of a redox-regulation circuit which regulates integrin α7β1 via its hinge-located thiol switch and thus affects initial steps of integrin-mediated cell adhesion (Figure 9c). Extracellular Trx1 and H_2_O_2_ activate and attenuate, respectively, integrin-binding activity. Such alternating changes of high and lower ligand-binding activities of integrins likely are necessary for migration, where cells attach at the front and detach at the rear. Thus, the local abundance and ratio of these oxidizing and reducing agents in the extracellular space redox-regulate integrins with functional consequences on their force exertion and cell migration. Worthwhile in this respect, the α7hiβ1-expressing cells do not form force-transmitting lamellipodia but bleb-like cell protrusions on laminin-111, implying that redox-regulated integrin activity might even change the mode of cellular migration.

## 5. Conclusions

Here, we showed that, in contrast to the structurally relevant disulfide bonds, the cysteine pair Cys604-Cys610 within the integrin α7 hinge region is a reversible thiol-switch, which determines the conformation and laminin-binding activity of integrins on both the molecular and cellular level. It can be regulated by micromolar concentrations of H_2_O_2_ and by extracellular Trx1, both of which are cell-produced mediators of redox regulation and thus signaling molecules for integrin-mediated cell migration. This may be a physiological redox-regulation circuit on the surface of cells during cell attachment and detachment, two basic steps in cell migration, where the integrin ectodomains mediate the force-transmitting and signal-conveying interaction with the extracellular matrix.
